# Clinical significance of *TP53* variants as possible secondary findings in tumor-only next-generation sequencing

**DOI:** 10.1038/s10038-019-0681-6

**Published:** 2019-10-18

**Authors:** Yoshihiro Yamamoto, Masashi Kanai, Tadayuki Kou, Aiko Sugiyama, Eijiro Nakamura, Hidehiko Miyake, Takahiro Yamada, Masakazu Nishigaki, Tomohiro Kondo, Hiromi Murakami, Masako Torishima, Shigemi Matsumoto, Shinji Kosugi, Manabu Muto

**Affiliations:** 1 Department of Medical Oncology, Graduate School of Medicine, Kyoto University, Kyoto, Japan; 2 DSK Project, Medical Innovation Center, Graduate School of Medicine, Kyoto University, Kyoto, Japan; 3 Faculty of Core Research, Natural Science Division, Ochanomizu University, Tokyo, Japan; 4 Clinical Genetics Unit, Kyoto University Hospital, Kyoto, Japan; 5 Department of Medical Ethics and Medical Genetics, Kyoto University School of Public Health, Kyoto, Japan; 6 Department of Human Health Sciences, School of Medicine, Kyoto University, Kyoto, Japan

**Keywords:** Cancer genomics, Targeted therapies, Cancer genetics

## Abstract

In tumor-only next-generation sequencing (NGS), identified variants have the potential to be secondary findings (SFs), but they require verification through additional germline testing. In the present study, 194 patients with advanced cancer who underwent tumor-only NGS between April 2015 and March 2018 were enrolled, and the incidences of possible and true SFs were evaluated. Among them, 120 patients (61.9%) harbored at least one possible SF. *TP53* was the most frequent gene in which 97 variants were found in 91 patients (49.5%). Nine patients provided informed consent to undergo additional germline testing, and a total of 14 variants (*BRCA1*, *n* = 1; *BRCA2*, *n* = 2; *PTEN*, *n* = 2; *RB1*, *n* = 1; *SMAD4*, *n* = 1; *STK11*, *n* = 1; *TP53*, *n* = 6) were analyzed. Three variants (*BRCA1*, *n* = 1; *BRCA2*, *n* = 2) were confirmed to be SFs, whereas *TP53* variants were confirmed to be somatic variants. To confirm the low prevalence of SFs in *TP53*, we analyzed 24 patients with *TP53* variants who underwent a paired tumor–normal NGS assay. As expected, all *TP53* variants were confirmed to be somatic variants. A total of 30 patients were tested for germline variants in *TP53*, but none of them resulted in true SFs, suggesting the low prevalence of SFs in this gene. Therefore, the significance of additional germline testing for *TP53* variants appears to be relatively low in daily clinical practice using a tumor-only NGS assay, unless patients have any relevant medical or family history.

## Introduction

Next-generation sequencing (NGS)-based multiplex gene assays are utilized to identify variants that predict the response or resistance to a specific drug in patients with cancer, and its use is rapidly increasing in daily clinical practice [1–6]. In addition, NGS assays can potentially uncover pathogenic germline variants, referred to as incidental or secondary findings (SFs) [7, 8]. The American College of Medical Genetics and Genomics (ACMG) has issued recommendations for the reporting of SFs to patients relating to 59 genes responsible for several hereditary diseases [8, 9]. The French Society of Predictive and Personalized Medicine has also published guidelines for the reporting of SFs relating to 60 cancer-related genes [9].

In a paired tumor–normal NGS assay, germline variants can be identified independently. Previous studies have reported that the prevalence of SFs in paired tumor–normal sequencing assays ranged between 3.3 and 17.5% [10–15]. NGS assays using only tumor DNA (tumor-only sequencing) are more commonly used in clinical practice to search for somatic mutations in tumor tissues, but they can also identify possible germline variants that can lead to SFs [1, 3, 6, 16]. However, to confirm the presence of true SFs, additional genetic testing using a matched germline DNA is necessary, and this verification process requires additional informed consent, cost, time and effort [16, 17].

The tumor suppressor *TP53* is the most commonly mutated gene in several types of cancer. Germline mutations in this gene are also known to be responsible for a rare hereditary disease, Li–Fraumeni syndrome (LFS). In tumor-only NGS assays, numerous *TP53* mutations can be identified; however, their origins are not always confirmed by additional germline analysis, as it requires substantial time and effort. Therefore, the clinical significance of this germline verification has not been evaluated in detail.

In the present study, we evaluated the incidence of possible SFs in ACMG-recommended genes in a tumor-only NGS assay and the clinical significance of additional germline testing to verify true SFs. Furthermore, we analyzed the data of 50 patients who underwent tumor–normal NGS assays and integrated data to assess the prevalence of germline variants in *TP53*.

## Methods

### Patient population

Between April 2015 and March 2018, a total of 194 patients with histopathologically confirmed solid tumors underwent a tumor-only NGS-based multiplex gene assay (OncoPrime^™^) at Kyoto University Hospital (Kyoto, Japan). In addition, a total of 50 patients underwent a paired tumor-only NGS assay, of which 30 patients also underwent the tumor-only NGS assay mentioned above (Supplementary Fig. 1). These assays were performed in patients with cancer of unknown primary site, rare tumors, and solid tumors refractory to standard chemotherapy. The present study was approved by the Ethics Committee of the Kyoto University Graduate School of Medicine (Kyoto, Japan; G692 and G1005). All patients provided written informed consent for the use of genomic and clinical data for research purposes.

### Patient medical and family history

Definite information regarding patient medical history and family histories of cancer were used to characterize the patients. Medical and family histories of LFS were assessed for patients with possible germline variants in *TP53* in accordance with either the classical LFS criteria or the Chompret criteria [18]. LFS-related cancer was defined as including sarcoma, breast cancer, brain cancer, adrenocortical carcinoma, leukemia, and lung cancer. Medical and family histories of hereditary breast and ovarian cancer (HBOC) were assessed for patients with possible germline variants in *BRCA1* or *BRCA2* in accordance with the genetic risk criteria, which were based on the National Comprehensive Cancer Network (NCCN) Clinical Practice Guidelines in Oncology, Genetic/Familial High-Risk Assessment: Breast and Ovarian, version 1, 2018. HBOC-related cancers were defined as including breast cancer, ovarian cancer, prostate cancer, and pancreatic cancer.

### Tumor-only NGS assay

OncoPrime^™^ is a tumor-only NGS-based multiplex gene assay, which uses only tumor DNA for sequencing [6]. This assay is designed to scan mutations and small insertions and deletions in 215 cancer-related genes and structural rearrangements in 17 genes with clinical or preclinical relevance in human solid tumors (Supplementary Table 1). NGS and data analysis were performed as previously described [6]. After the NGS assay was ordered by the treating physician, 5–10 slices of 10 μm sections of archival formalin-fixed paraffin-embedded (FFPE) tumor tissue (tumor content ≥20%) were sent to a Clinical Laboratory Improvement Amendment (CLIA)-certified Laboratory of EA Genomics (Morrisville, NC, USA) and DNA extraction was performed by EA Genomics. The DNA extracted from fresh-frozen tumor tissues at our institution was sent to the Laboratory of EA Genomics. Solution hybridization targeted 3861 exons of 215 cancer-related genes and 59 introns of 17 genes commonly rearranged in human cancers using SureSelect XT reagent (Agilent Technologies, Santa Clara, CA, USA). Sequencing was performed using the Illumina HiSeq 2500 system.

### Paired tumor–normal NGS assay

The NGS-based analysis of matched tumor–normal pairs utilized the National Cancer Center oncopanel v4, which analyzed 114 cancer-associated genes (Supplementary Table 2) [19]. The NGS assay and data analysis were performed in the CLIA-certified and College of American Pathologist-accredited Laboratory of Riken Genesis Co., Ltd. (Tokyo, Japan). Sections of archival FFPE tumor tissue (tumor content ≥20%) and matched blood sample were sent to the Laboratory of Riken Genesis, where DNA extraction was performed. The DNA extracted from FFPE or fresh-frozen tumor tissues at our institution was sent to the Laboratory of Riken Genesis. NGS libraries were prepared using SureSelect XT reagent (Agilent Technologies, Santa Clara, CA, USA) and the KAPA Hyper Prep kit (KAPA Biosystems, Wilmington, MA, USA). Sequencing was performed using MiSeq or NextSeq platform (Illumina, San Diego, CA, USA). Sequencing data were analyzed to detect somatic and germline variants independently, as described previously [19].

### Determination of the pathogenicity of variants

The pathogenicity of variants was determined predominantly on the basis of the clinical data reported in ClinVar (http://www.ncbi.nlm.nih.gov/clinvar/). Nonsense and frameshift mutations that were not registered in ClinVar were determined as pathogenic if there were one or more known pathogenic truncated variants in the posterior region to the sites. Variants in a splice site that were not registered in ClinVar were determined as likely pathogenic if there were known pathogenic variants in the same splice site. Variants registered as uncertain significance (VUS) or conflicting interpretations of pathogenicity were classified as likely pathogenic if pathogenic or likely pathogenic reports were predominant in ClinVar. If there was no significant information on the pathogenicity of variants in ClinVar, the following public databases were also referred to: Catalog of Somatic Mutations in Cancer (COSMIC) (http://cancer.sanger.ac.uk/cosmic), Insight (https://www.insight-group.org), Leiden Open Variation Database (LOVD) (http://www.lovd.nl/3.0/home), and the University of Utah Department of Pathology and ARUP Laboratories database (http://arup.utah.edu/database/BRCA/). For *TP53* variants, a functional classification based on its translational activity in the International Agency for Research on Cancer (IARC) TP53 Database (http://p53.iarc.fr/) was also referred to.

### Germline analysis using Sanger sequencing

**Fig. 1:**
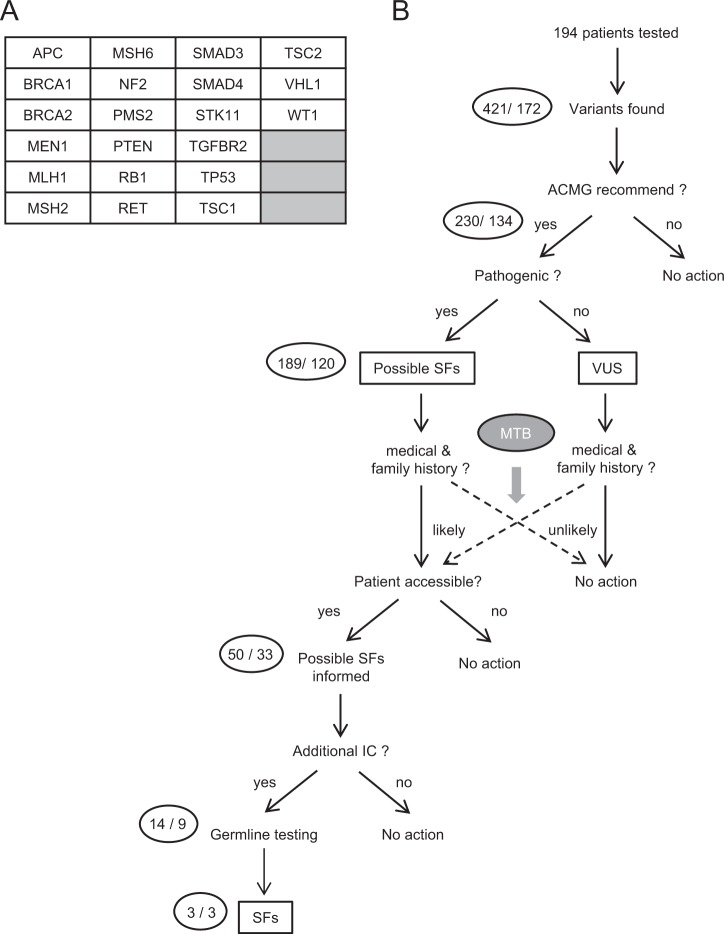
Verification of secondary findings in the study using a tumor-only next-generation sequencing (NGS) assay. **a** Set of target genes in the tumor-only NGS also included in the ACMG recommendation. **b** In a study using the tumor-only NGS assay, pathogenic or likely-pathogenic variants in ACMG-recommended genes were defined as possible secondary findings (SFs). The necessity of germline testing for possible SFs was carefully discussed in molecular tumor board (MTB). Variants of uncertain significance (VUS) were included in subjects undergoing germline testing if medical and family histories of the patients were more evident. Possible SFs were informed only to patients for whom our institute was accessible to propose additional germline testing. Patients who provided additional informed consent underwent blood sampling and germline testing. The number of variants and patients are shown in the circle on the left of each step (variants/patients)

**Fig. 2:**
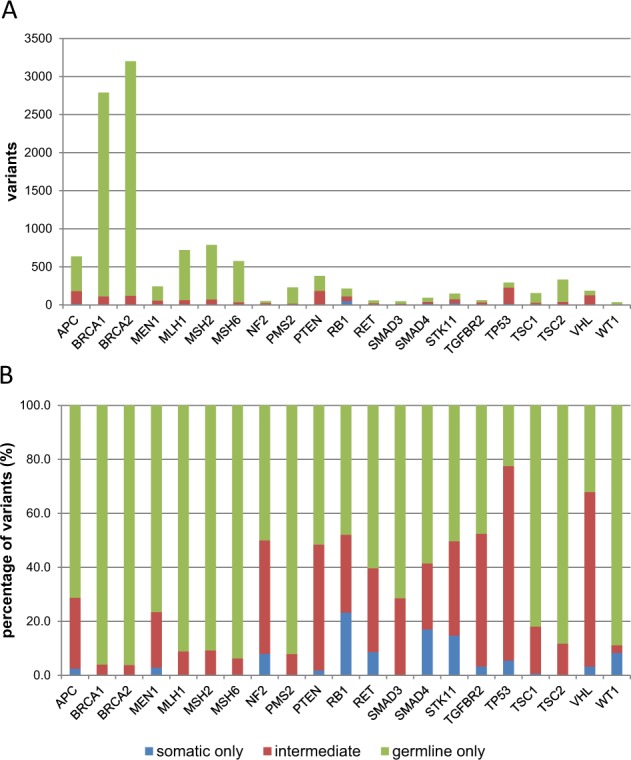
Distribution of known somatic and germline variants in 21 genes included in the ACMG recommendation. Pathogenic or likely pathogenic variants in ACMG-recommended genes were extracted from the ClinVar database and were divided into three groups: somatic-only, germline-only, and intermediate. **a** Variant count. **b** Percentage of variants (%)

Following the tumor-only NGS assay, germline analyses were performed in patients meeting the following criteria: (1) harboring at least one possible germline variant, including pathogenic or likely pathogenic variants, VUSs and unknown variants in ACMG-recommended genes (Fig. 1a), (2) having suspected medical and family histories of cancer, and (3) agreeing with and providing written informed consent for additional germline analysis (Fig. 1b).

Germline DNA was extracted from the peripheral blood of patients using the GENE PREP STAR NA-480 system (KURABO Industries, Ltd., Osaka, Japan) in the Cancer BioBank of Kyoto University Hospital. The genomic fragments comprising the region of the variant were amplified by polymerase chain reaction using PrimeSTAR MAX (Takara Bio, Inc., Tokyo, Japan) and were used as templates for Sanger sequencing, which was performed by Eurofins Genomics Co., Ltd. (Tokyo, Japan).

### Molecular tumor board

Our institutional molecular tumor board comprises several medical professionals, including medical oncologists, pathologists, medical geneticists, genetic counselors, genomic researchers, bioinformaticians, pharmacists, nurses, and clinical research coordinators. The molecular tumor board meets weekly to discuss genetically informed treatment options and the possibility of SFs.

### Variant database analysis

The pathogenic and likely-pathogenic variants downloaded from the ClinVar database were filtered to eliminate structural variants, variants with ambiguous information, and variants mapped on minor transcripts. Variants that were registered only as somatic in ClinVar were classified as “somatic-only” variants, whereas those that were registered only as germline in ClinVar and were not registered in COSMIC were classified as “germline-only” variants. The remaining variants were classified as “intermediate” variants, which included the following: (a) variants that were registered both as somatic and germline in ClinVar or (b) variants that were registered as germline in ClinVar and also registered as somatic in COSMIC. Data on the variants were independently analyzed for each ACMG gene.

## Results

### Incidence of possible germline variants in the tumor-only NGS assay

Of the 194 patients who underwent the tumor-only NGS assay, OncoPrime^™^, 120 patients (61.9%) were found to harbor at least one pathogenic or likely pathogenic variant in ACMG-recommended genes, which could be classified as possible SFs (Fig. 1). The characteristics of patients with possible SFs are summarized in Table 1, in which colorectal cancer (*n* = 24) was the most common type of cancer and pancreatic cancer (*n* = 21) was the second most common. The most commonly mutated gene in these possible SFs was *TP53* (*n* = 97), followed by *APC* (*n* = 38) and *PTEN* (*n* = 11) (Table 2). There was a total of 13 possible SFs in *BRCA1* (*n* = 5) and *BRCA2* (*n* = 8) combined. The medical and family histories of LFS- and HBOC-related cancer were summarized for patients carrying possible SFs in *TP53* and *BRCA1/2*, respectively (Table 3). None of the patients had a medical or family history suggestive of LFS, although 91 patients (46.9%) were identified to have one or more possible SFs in *TP53*.

**Table 1:** Characteristics of patients undergoing the tumor-only NGS assay and germline verification

Characteristics	No. of patients (%)		
	With possible SFs (*n* = 120)	Tested by Sanger (*n* = 9)	With confirmed SFs (*n* = 3)
Age			
<60 years	70 (58.3)	5 (55.6)	0 (0)
≥60 years	50 (41.7)	4 (44.4)	3 (100)
Sex			
Male	53 (44.2)	6 (66.7)	2 (66)
Female	67 (55.8)	3 (33.3)	1 (33)
Cancer type			
Colorectal	24 (20.0)	1 (11.1)	0 (0)
Pancreas	21 (17.5)	2 (22.2)	1 (33)
Unknown primary	11 (9.2)	0 (0)	0 (0)
Gastric	10 (8.3)	2 (22.2)	2 (66)
Esophagus	9 (7.5)	1 (11.1)	0 (0)
Ovary	9 (7.5)	1 (11.1)	0 (0)
Biliary tract	8 (6.7)	0 (0)	0 (0)
Breast	6 (5.0)	0 (0)	0 (0)
Neuroendocrine	5 (4.2)	0 (0)	0 (0)
Uterine and cervical	4 (3.3)	1 (11.1)	0 (0)
Urinary tract	4 (3.3)	1 (11.1)	0 (0)
Liver	3 (2.5)	0 (0)	0 (0)
Head and neck	3 (2.5)	0 (0)	0 (0)
Lung	1 (0.8)	0 (0)	0 (0)
Bone and soft tissue	1 (0.8)	0 (0)	0 (0)
Brain	1 (0.8)	0 (0)	0 (0)
Family history of any cancers			
Both in FDR/SDR	13 (10.8)	1 (11.1)	0 (0)
Only in FDR	37 (30.8)	6 (66.7)	3 (100)
Only in SDR	10 (8.3)	0 (0)	0 (0)

*SFs* secondary findings, *FDR* first degree relatives, *SDR* second degree relatives

**Table 2:** Possible and confirmed secondary findings in 21 genes included in the ACMG recommendation both in tumor-only and tumor–normal NGS assays

Gene name	No. of variants/patients						
	Tumor-only NGS assay			Tumor–normal NGS assay		Summary of confirmed variants	
	Possible SFs	Tested by Sanger	Confirmed SFs	Somatic variants	SFs	Somatic variants	SFs
*TP53*	97/91	6/6	0/0	24/24	0/0	30/30	0/0
*APC*	38/28	0/0	0/0	9/7	0/0	9/7	0/0
*PTEN*	11/7	2/1	0/0	0/0	0/0	2/1	0/0
*RB1*	9/8	1/1	0/0	5/5	0/0	6/6	0/0
*SMAD4*	9/9	1/1	0/0	3/3	0/0	4/4	0/0
*BRCA2*	8/7	2/2	2/2	1/1	0/0	1/1	2/2
*BRCA1*	5/5	1/1	1/1	0/0	0/0	0/0	1/1
*STK11* (VUS included)	4/3	0/0 (1/1)	0/0	1/1	0/0	1/1 (2/2)	0/0
*MEN1*	2/2	0/0	0/0	–	–	0/0	0/0
*MLH1*	1/1	0/0	0/0	0/0	0/0	0/0	0/0
*MSH2*	1/1	0/0	0/0	0/0	0/0	0/0	0/0
*PMS2*	1/1	0/0	0/0	–	–	0/0	0/0
*TSC1*	1/1	0/0	0/0	0/0	0/0	0/0	0/0
*TSC2*	1/1	0/0	0/0	–	–	0/0	0/0
*RET, VHL*	0/0	0/0	0/0	0/0	0/0	0/0	0/0
*MSH6, NF2, SMAD3, TGFRB2, WT1*	0/0	0/0	0/0	–	–	0/0	0/0
Total	189/120	13/8 (14/9)	3/3	43/37	0	53/39 (54/40)	3/3

*SFs* secondary findings

**Table 3:** Medical and family history of LFS- or HBOC-related cancers in patients undergoing tumor-only NGS assay

Medical and family history of LFS-related cancers^a^	No. of patients with variants in *TP53* (%)		
	With possible SFs (*n* = 91)	Tested SangerSeq (*n* = 6)	With true SFs (*n* = 0)
In			
P + FDR + SDR	0 (0)	0 (0)	0 (–)
P + FDR	4 (6.1)	0 (0)	0 (–)
P + SDR	0 (0)	0 (0)	0 (–)
P	2 (3.0)	0 (0)	0 (–)
FDR + SDR	0 (0)	0 (0)	0 (–)
FDR	4 (6.1)	0 (0)	0 (–)
LFS criteria matched	0 (0)	0 (0)	0 (–)

Medical and family history of HBOC-related cancers^b^	No. of patients with variants in *BRCA1* or *BRCA2* (%)		
	With possible SFs (*n* = 11)	Tested SangerSeq (*n* = 3)	With true SFs (*n* = 3)
In			
P + FDR + SDR	0 (0)	0 (0)	0 (0)
P + FDR	1 (9.1)	0 (0)	0 (0)
P + SDR	0 (0)	0 (0)	0 (0)
P	5 (45.5)	1 (33.3)	1 (33.3)
FDR + SDR	0 (0)	0 (0)	0 (0)
FDR	1 (9.1)	1 (33.3)	1 (33.3)
HBOC criteria matched	1 (9.1)	0 (0)	0 (0)

^a^Sarcoma, breast cancer, brain tumor, adrenocortical carcinoma, leukemia, or lung cancer

^b^Breast cancer, ovarian cancer, prostate cancer, or pancreatic cancer

*SFs* secondary findings, *LFS* Li–Fraumeni syndrome, *HBOC* hereditary breast and ovarian cancer, *P* patient, *FDR* first degree relatives, *SDR* second degree relatives

### Verification of SFs

As shown in Fig. 1, 9 patients agreed to the additional germline testing, and a total of 14 possible germline variants were examined by Sanger sequencing using the germline DNA samples. Following this verification, SFs were confirmed in three patients, in which pathogenic germline variants in *BRCA1* (Q934*) or *BRCA2* (R2318* and Q3026*) were confirmed (Table 4 and Supplementary Fig. 2), although these patients had no evident medical or family history of HBOC (Table 3). In accordance with the recommendations of the ACMG, the patients were carefully informed of these SFs in *BRCA1* and *BRCA2* by medical experts in genetic counseling. By contrast, all six variants tested in *TP53* were confirmed as somatic variants (Tables 2 and 4 and Supplementary Fig. 1).

**Table 4:** Summary of patients undergoing germline verification using Sanger sequencing

Patient	Cancer type	Age	Sex	Family history	Gene	Variant	ClinVar	VAF	SangerSeq
1	Rectal AC	57	Male	–	*TP53*	G245D	Pathogenic	0.48	Negative
2	Gastric AC	82	Male	Esophageal (F)	*BRCA1* *TP53*	Q934* R175H	Pathogenic Pathogenic	0.69 0.44	Positive Negative
3	Pancreatic AC	47	Male	Pancreatic (F), Pancreatic (M)	*SMAD4* *TP53*	Q448* T125R	Pathogenic Conflicting	0.62 0.62	Negative Negative
4	Pancreatic AC	68	Female	Gastric (M)	*BRCA2* *TP53*	R2318* Q165*	Pathogenic Pathogenic	0.44 0.29	Positive Negative
5	Esophageal SCC	62	Male	Pancreatic (F), Colon (M)	*TP53*	W91*	Pathogenic	0.21	Negative
6	Uterine AC	48	Female	Bile duct (F), Lymphoma (GF), Lung (GM)	*STK11*	H174Y	Uncertain significance	0.40	Negative
7	Gastric AC	74	Male	Pancreatic (B)	*BRCA2* *TP53*	Q3026* c.783-1 G > T	Pathogenic –	0.48 0.22	Positive Negative
8	Urothelial carcinoma	64	Male	Malignant tumor (M)	*RB1*	Q62*	–	0.38	Negative
9	Ovarian AC	41	Female	–	*PTEN* *PTEN*	C136Y D51fs	Pathogenic –	0.29 0.28	Negative Negative

*AC* adenocarcinoma, *SCC* squamous cell carcinoma, *VAF* variant allele frequency, *F* father, *M* mother, *GF* grandfather, *GM* grandmother, *B* brother

### Variant findings in *TP53* using paired tumor–normal sequencing

To confirm the low prevalence of true SFs in *TP53*, we analyzed data from 50 cases of paired tumor–normal NGS assay, of which 30 patients underwent a tumor-only NGS assay in parallel (Supplementary Fig. 1). In those 50 patients, 24 pathogenic or likely pathogenic variants in *TP53* were identified; however, none of them were germline variants (Table 2, Supplementary Fig. 1 and Supplementary Table 3). Taken together with results of Sanger sequencing, a total of 30 variants in *TP53* were confirmed as somatic variants (Table 2 and Supplementary Tables 3 and 4). These results indicate that the incidence of true SFs in *TP53* is low, despite being the most commonly detected possible germline variant in the tumor-only NGS assay.

### Classification of known variants in ACMG-recommended genes

Subsequently, we examined the proportion of somatic and germline variants registered in the ClinVar database for *TP53* and another 20 ACMG-recommended genes that were included in our tumor-only NGS panel. All variants were classified into somatic-only, germline-only, and intermediate, and their numbers were counted for each gene to compare them (Fig. 2a). The proportion of somatic variants in *TP53* was 77.6%, and the majority of these overlapped with germline variants (intermediate variants, 72.1%; Fig. 2b). By contrast, somatic-only or intermediate variants were rare in *BRCA1* and *BRCA2* (4.0% and 3.8%, respectively; Fig. 2b).

## Discussion

In the present study, 120 patients (61.9%) harbored at least one possible germline variant; of these patients, nine underwent additional germline testing to determine whether these variants were true SFs. The results confirmed that three patients had SFs, the proportion of which was equivalent to 1.5% of total patients and 33% of germline-verified patients. The possible reasons for many patients not undergoing additional germline testing were as follows: (1) deterioration in their physical conditions, preventing sufficient time for either patients or physicians to discuss the issue of SFs; (2) poor accessibility of the patients to our hospital to participate in the germline testing; and (3) the burden of undergoing additional germline testing to patients, despite them being concerned about possible SFs.

Considering the confirmation of SFs, a paired tumor–normal NGS assay has a substantial advantage over a tumor-only NGS assay, as the former assay does not require additional germline testing [16]. A previous study using a paired tumor–normal NGS assay revealed that the prevalence of SFs was between 3.3 and 17.5% [10–15]. Because only 7.5% of cases with possible germline variants actually underwent additional germline testing, it is likely that the prevalence of SFs is underestimated in the present study.

The NCCN guidelines of HBOC recommend a genetic testing of *BRCAs* for patients who meet the criteria such as medical history of bilateral breast cancer, triple-negative breast cancers or ovarian cancer and family history of related cancers. However, in the three patients confirmed to have SFs in *BRCA1/2* in our present study, none reported a typical medical and family history of HBOC. This suggested that consideration of medical and family histories is insufficient for the prediction of germline findings in *BRCA1/2*. We also emphasize that the majority of the known variants in *BRCA1* and *BRCA2* have been registered as germline variants only (96% each) (Fig. 2). In addition, previous genome profiling study against advanced cancers indicated that more than 75% of pathogenic *BRCA1/2* variants identified in tumors were originated from germline variants [11]. Based on these data, if any pathogenic or likely pathogenic variant is found in *BRCA1/2* using a tumor-only NGS assay, additional germline testing is recommended.

In contrast to the above, the prevalence of true SFs in *TP53* appears to be low, despite being the most commonly detected possible SF (75.8%) in the tumor-only NGS assay. Although just 6.6% (6/91) of possible SFs in *TP53* were verified in Sanger sequencing of germline samples, none of them were found to be true SFs (Tables 2 and 4). In addition, no SFs were identified in *TP53* using the paired tumor–normal NGS assays in 50 patients, whereas 24 patients were identified to have pathogenic or likely pathogenic somatic variants in *TP53* (Table 2 and Supplementary Table 3). Supporting our observations, the incidence of SFs in *TP53* has been reported to be 0–1% using paired tumor–normal assays [10–15].

It has been reported that somatic hotspot mutations in *TP53* identified in various types of tumor overlap with those in germline variants [20]. We performed analysis using a public database and revealed that >70% of pathogenic or likely pathogenic variants in *TP53* were registered as both somatic and germline variants, which was the highest coincidence among the 21 genes recommended by the ACMG in our tumor-only NGS panel (Fig. 2). This coincidence of somatic and germline mutations appears to increase the difficulty in distinguishing true SFs in *TP53* from the numerous somatic variants when they were identified in the tumor-only NGS assay.

*TP53* has been known as the gene causing LFS, a rare hereditary cancer syndrome characterized by the early onset of various types of cancer, including sarcoma, adrenocortical carcinoma, breast cancer, leukemia, and brain tumors [20, 21]. It is reported that >80% of *TP53* mutation carriers meet the diagnostic criteria for LFS or Li–Fraumeni-like criteria, having a family history suggestive of and/or early onset of LFS-related cancer [18, 22]. It has also been reported that there are de novo mutations in *TP53*, which are relatively frequent in patients who have early onset LFS-related cancer in the absence of family history [23, 24]. Therefore, the medical and family histories of a patient serve an important role in determining whether to proceed with additional germline testing when possible SFs in *TP53* are identified using a tumor-only NGS assay. This is consistent with a recent publication by the European Society of Medical Oncology Precision Medicine Working Group, which recommends germline testing for patients with suspected *TP53* germline mutations only if those associated tumors arise at <30 years of age [25].

In conclusion, the tumor-only NGS assay yielded a number of possible SFs in *TP53*; however, the significance of additional germline testing for *TP53* variants appears fairly low in daily clinical practice using a tumor-only NGS assay, unless patients report any relevant medical or family histories.

## Supplementary information

Supplementary Fig. 1

Supplementary Fig. 2

Supplementary Table 1

Supplementary Table 2

Supplementary Table 3

Supplementary Table 4
