# Supplementary material for: Clinical significance of TP53 variants as possible secondary findings in tumor-only next-generation sequencing
Source: J Hum Genet. 2019 Oct 18;65(2):125–32. doi: 10.1038/s10038-019-0681-6 (PMC6917569; doi:10.1038/s10038-019-0681-6)
Supplement: Supplementary file 1 — Supplementary Fig. 1 [file 10038_2019_681_MOESM1_ESM.pptx]

## Slide 1
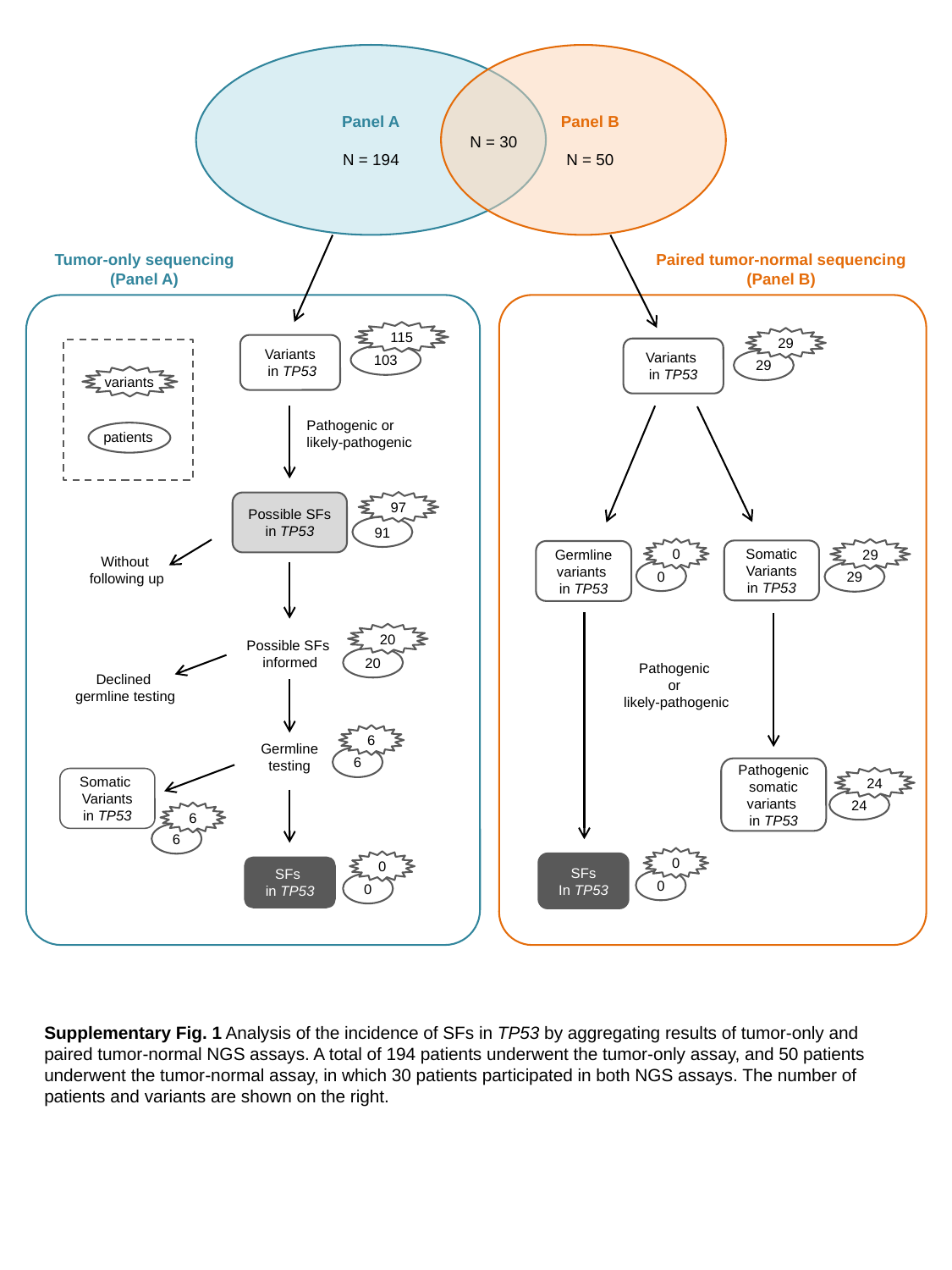

Panel A
N = 194
 Panel B
 N = 50
N = 30
Tumor-only sequencing
(Panel A)
Paired tumor-normal sequencing
(Panel B)
115
29
Variants
 in TP53
Variants
in TP53
103
29
variants
Pathogenic or likely-pathogenic
patients
97
Possible SFs
in TP53
91
0
29
Somatic Variants in TP53
Germline variants
in TP53
Without
following up
0
29
20
Possible SFs informed
20
Pathogenic
or
likely-pathogenic
Declined
germline testing
6
Germline testing
6
Pathogenic somatic variants
in TP53
24
Somatic
Variants in TP53
24
6
6
0
0
SFs
In TP53
SFs
in TP53
0
0
Supplementary Fig. 1 Analysis of the incidence of SFs in TP53 by aggregating results of tumor-only and paired tumor-normal NGS assays. A total of 194 patients underwent the tumor-only assay, and 50 patients underwent the tumor-normal assay, in which 30 patients participated in both NGS assays. The number of patients and variants are shown on the right.
