# Supplementary material for: Clinical significance of TP53 variants as possible secondary findings in tumor-only next-generation sequencing
Source: J Hum Genet. 2019 Oct 18;65(2):125–32. doi: 10.1038/s10038-019-0681-6 (PMC6917569; doi:10.1038/s10038-019-0681-6)
Supplement: Supplementary file 2 — Supplementary Fig. 2 [file 10038_2019_681_MOESM2_ESM.pptx]

## Slide 1
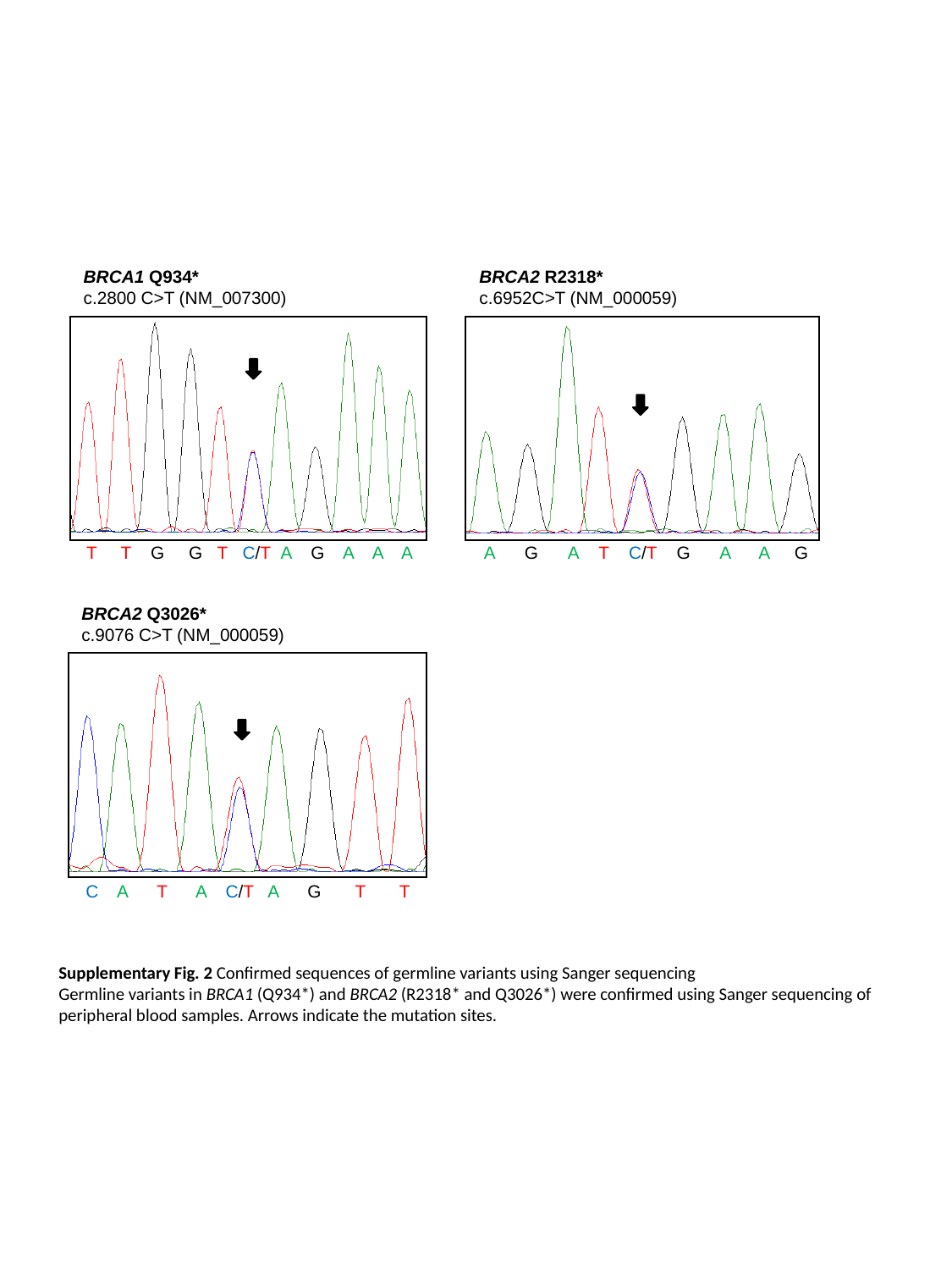

BRCA1 Q934*
c.2800 C>T (NM_007300)
BRCA2 R2318*
c.6952C>T (NM_000059)
T T G G T C/T A G A A A
A G A T C/T G A A G
BRCA2 Q3026*
c.9076 C>T (NM_000059)
C A T A C/T A G T T
Supplementary Fig. 2 Confirmed sequences of germline variants using Sanger sequencing
Germline variants in BRCA1 (Q934*) and BRCA2 (R2318* and Q3026*) were confirmed using Sanger sequencing of peripheral blood samples. Arrows indicate the mutation sites.
